# Working together for the family: determination of HER oncogene co-amplifications in breast cancer

**DOI:** 10.18632/oncotarget.27671

**Published:** 2020-07-14

**Authors:** Sergio Laurito, María Teresita Branham, Emanuel Campoy, Sebastián Real, Juan Cueto, Guillermo Urrutia, Francisco Gago, Olga Tello, Telma Glatstein, Paola De la Iglesia, Lilit Atanesyan, Suvi Savola, Maria Roqué

**Affiliations:** ^1^Institute of Histology and Embryology, National Council of Research, Consejo Nacional de Investigaciones Científicas y Técnicas, Mendoza, Argentina; ^2^Universidad Nacional de Cuyo, Facultad de Ciencias Exactas y Naturales, Mendoza, Argentina; ^3^Universidad Nacional de Cuyo, Facultad de Ciencias Médicas, Mendoza, Argentina; ^4^Division of Research, Department of Surgery, Medical College of Wisconsin, Milwaukee, WI, USA; ^5^Instituto Gineco-Mamario, Mendoza, Argentina; ^6^CARPAT SA, Mendoza, Argentina; ^7^Hospital Italiano, Servicio de Anatomía Patológica, Buenos Aires, Argentina; ^8^MRC-Holland BV, Department of Oncogenetics, Amsterdam, The Netherlands

**Keywords:** HER oncogenes, breast cancer, MLPA, digital PCR, co-amplification

## Abstract

HER2 is a well-studied tyrosine kinase (TK) membrane receptor which functions as a therapeutic target in invasive ductal breast carcinomas (IDC). The standard of care for the treatment of HER2-positive breast is the antibody trastuzumab. Despite specific treatment unfortunately, 20% of primary and 70% of metastatic HER2 tumors develop resistance. HER2 belongs to a gene family, with four members (HER1-4) and these members could be involved in resistance to anti-HER2 therapies. In this study we designed a probemix to detect the amplification of the four HER oncogenes in a single reaction. In addition, we developed a protocol based on the combination of MLPA with ddPCR to detect the tumor proportion of co-amplified HERs. On 111 IDC, the HER2 MLPA results were validated by FISH (Adjusted *r*^2^ = 0,91, *p* < 0,0001), CISH (Adjusted *r*^2^ = 0,938, *p* < 0,0001) and IHC (Adjusted *r*^2^ = 0,31, *p* < 0,0001). HER1-4 MLPA results were validated by RT-qPCR assays (Spearman Rank test *p* < 0,05). Of the 111 samples, 26% presented at least one HER amplified, of which 23% showed co-amplifications with other HERs. The percentage of cells with HER2 *co-amplified* varied among the tumors (from 2–72,6%). Independent *in-silico* findings show that the outcome of HER2+ patients is conditioned by the status of HER3 and HER4. Our results encourage further studies to investigate the relationship with patient’s response to single or combined treatment. The approach could serve as proof of principle for other tumors in which the HER oncogenes are involved.

## INTRODUCTION

The human epidermal growth factor receptor HER2 (ErbB2, HGNC: 3430) is a well-studied tyrosine kinase (TK) membrane receptor which functions as a therapeutic target in invasive ductal breast carcinomas (IDC). The over-expression of HER2 due to oncogene amplification occurs in up to 15–20% of IDC [1]. It is the only predictive biomarker in breast cancer indicative of targeted therapies with monoclonal antibodies. Today, the antibody trastuzumab remains the standard of care for the treatment of HER2-positive breast cancer in both, the early and advanced disease stage [2]. However, approximately a quarter of patients still relapse up to 10 years after diagnosis [3] even though trials such as the HERA and BCIRG 006 have introduced the benefit from adding adjuvant trastuzumab to chemotherapy [4, 5]. The individual response to anti-HER2 based therapies is still highly heterogeneous, since under *similar* conditions of HER2 some tumors present complete response while others do resist the treatment [6]. The differential response is unclear and probably due to a sum of multiple factors including genomic background (such as constitutive active PI3K/Akt pathway due to PTEN loss or PIK3CA activating mutations) and patient-specific features (for example, treatment with recombinant human eritropoyetin to manage treatment-induced anemia). But what seems clear is that it is not attributable only to the *amount* of HER2 protein, and probably the presence of *other* biomarkers is interfering in the efficacy of the therapy.

Gene family is a set of genes with a common ancestral origin, which participate typically in similar biological functions encoding for functionally related proteins. Some well-known examples are the homeobox gene family, myosin gene family and heat shock protein family. HER2 also belongs to a gene family (the HER receptor family), with four members (HER1: EGFR, HGNC:3236-; HER2: ErbB2, HGNC:3430; HER3: ErbB3, HGNC:3431; HER4: ErbB4, HGNC:3432).

The four HER proteins are membrane bound TK receptors and form homo and heterodimers with each other after ligands binding [7], revealing a synergic functioning. In fact, two of the members-HER2 and HER3- are non-autonomous, because HER2 lacks ligand-dependent activation and HER3 has no TK activity. Consequently, both need other members to activate their oncogenic capacity. In a non-tumoral scenario HER receptors exist as inactive monomers with the molecules folded to avoid possible dimerization [8]. In cancer, however, the four receptors have an important role in enhancing cell proliferation, mainly by increasing the downstream signaling of ERK1/2 and PI3K/Akt and promote cell survival, angiogenesis, and metastasis [9]. The HER family is a robust redundant network which regulates characteristic functions of the tumorigenic process i.e. proliferation, invasiveness and survival. If one member is downregulated, the others are capable to compensate and continue downstream the signaling to the nucleus. As described by Citri et al. [10], the HER family functions in a bow-tie architecture. This means that the inputs of different ligands interact with potentially homo or heterodimerized HER receptors, activates a *small* core set of molecules that regulate *multiple* outputs. This type of functioning is robust and resistant to common perturbations, where the main advantage is the dynamism and plasticity. In this bow-tie context, HER2 is the favorite dimerization-partner and the strongest positive regulator. In accordance, a decade ago HER2 was proposed as the master coordinator of the HER oncogene family [11]. Therefore, it is reasonable to expect that the members of the same family could be the natural candidates to confer part of the resistance to anti-HER2 therapies, as has already been proposed by others [12].

In this work we aimed to develop an original experimental approach to study simultaneously the gene amplification of all the HER family members in breast tumors and to determine the percentage of a tumor in which co-amplifications occur in the same cell. We choose the methodology MLPA as it permits simultaneous detection of copy number changes on different genes; it is affordable as well as easy to use on Formalin-fixed paraffin-embedded tissues (FFPE) samples. To this end, we have generated a MLPA probemix for the four HER genes and validated it by FISH, CISH, IHC and RT-qPCR. To distinguish “normal” from “amplified” status, we propose a cut-off value for each HER, as there is a lack of a clinically validated scoring algorithm for HER1, HER3 and HER4 in breast tumors. Further on, while many studies report elevated expression or amplification of individual members of the HER family, there are limited studies, as far as we know, evaluating their *co*-expression or amplification in the same cell. So, to accomplish this, we propose the utilization of the MLPA kit in a partitioning digital PCR system to determine the number of cells with co-amplifications of HER2 with other HER members. A similar strategy was attempted to evaluate the expression of dual-target detection in single bacterial cells using ddPCR [13], but to the best of our knowledge our experimental approach is the first to combine MLPA and ddPCR system to detect HER family amplification. We show by this algorithm that HER co-amplifications do occur, and that this phenomenon is highly heterogeneous in the tumor cell population of the studied breast tumors.

## RESULTS

### MLPA probe mix development to assess the amplification of 4 HER oncogenes

The rationality of the probe design had to foresee the inclusion of probes targeting both, amino and carboxyl coding exons of the four HER genes, since it is known that partial HER amplifications can lead to the over-expression of truncated non-functional receptors [14]. For HER1 and HER3 there was an impediment, since their first exons present an enhanced GC content which interferes in the melting steps of the assay and are therefore not a reliable site for probe hybridization. To sort this out, probes for HER1 were designed to target at least an exon which coded for the extracellular domain and one for the TK domain. For HER3 only exons coding for the receptor domain were designed since it lacks TK intracellular domain. By this, the design attempted to provide genomic information which could allow further correlations with functional protein expression. The probes were manufactured as described by Schouten et al. [15] at MRC-Holland. The new kit contained six probes for HER1, eight for HER2, four for HER3 and five for HER4 (see details in Table 1), plus eight probes flanking the HER regions and 16 reference probes. The latter probemix was denominated as P483-A1 and used for further analyses.

**Table 1:** Description of MLPA probes, P483-A1 mix

Gene	Chromosome location	Total Number of exons	Number of probes per gene	Exon number where MLPA probes hybridize
EGFR (HER1)	7p11.2	28	6	2, 10, 11, 20, 23, 28
ERBB2 (HER2)	17q12	33	8	1, 8, 13, 14, 17, 24, 29, 32
ERBB3 (HER3)	12q13.2	30	4	3a/b, 11, 21, 25
ERBB4 (HER4)	2q34	28	5	1, 2, 3, 12, 20

Details of the designed MLPA probes for each HER oncogene.

### Validation of MLPA based HER2-determination by the gold-standard FISH, CISH, IHC assays and by qPCR

78 IDCs were included for the MLPA validation, of which 57 were embedded in paraffin blocks (FFPE) and used for a prospective validation, and 21 were frozen fresh tumor tissues and were included for a retrospective correlation. We based the validation on the determination of HER2, as there are gold-standard well-established procedures with international consensus in the guidelines developed by the American Society of Clinical Oncology (ASCO) and the College of American Pathologists (CAP) [1]. These guidelines suggest analyzing HER2 at protein level by IHC and at DNA level by genomic fluorescent/chromogenic *in situ* hybridization (FISH/CISH). Any new Laboratory Developed Test (LDT) should be validated in a prospective manner by the gold standard methodologies.

We examined 57 FFPE tumors following the ASCO/CAP recommendations as follows: i.e by IHC (*N* = 43) and CISH (*N* = 16) in a Ventana Benchmark System with external validation controls (i.e. Nordicq IHC Quality Control, https://www.nordiqc.org/about.php), and ii. by FISH (*N* = 40) in a Dako Hybridizer, with a validated protocol for patient diagnoses. In addition, we also tested the MLPA results including 21 fresh frozen tumors in which IHC data was obtained by in-house manual lab assay, this was done to increase the sample size (which we called *global study*, *N* = 78). Study design is represented in Supplementary Figure 1.

**Figure 1:**
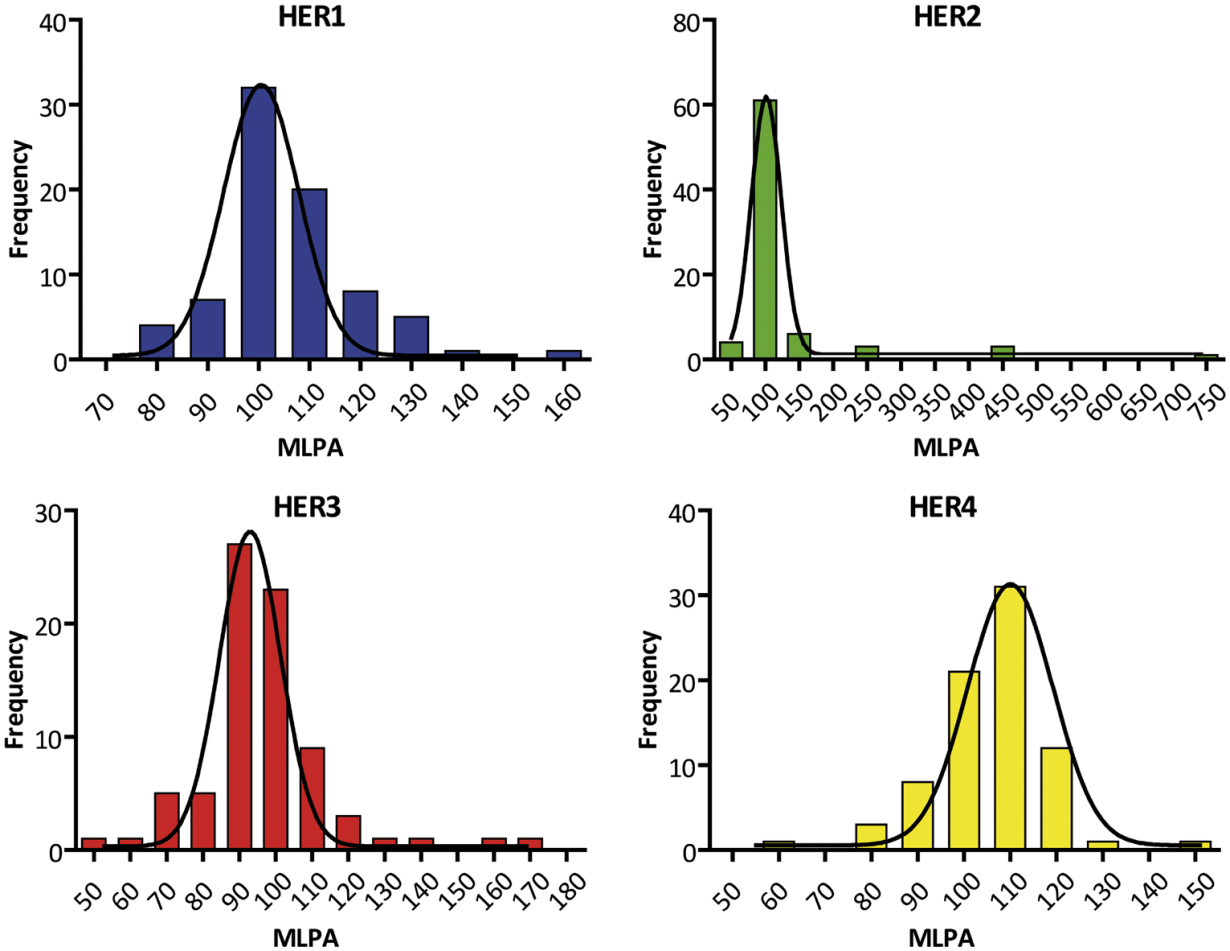
Frequency distribution of the MLPA ratios expressed in percentages of HER gene family members. The frequency distribution was obtained from 78 FFPE plus fresh IDCs. None passes the normality test (KS Test. *p* > 0.05). The descriptive statistics show highly different distributions (note the different scales in X axis). i.e : HER1: median = 103.91. mean = 105.94. SD = 13.56; HER2: median = 102. mean = 128.54. SD = 10.28; HER3: median = 95.12. mean = 96.53. SD = 18.40; HER4: median = 105. mean = 104.51. SD = 12.24.

As the P483-A1 probemix includes probes for several exons of the same HER, an average of the probe signals was calculated to define the value for each gene. A normalized ratio of the MLPA signal between tumor-sample/control-sample was determined for each probe and afterwards the mean of all probes was calculated for each gene. The frequency distribution of the MLPA results in the studied tumors is shown in Figure 1.

**Figure 2:**
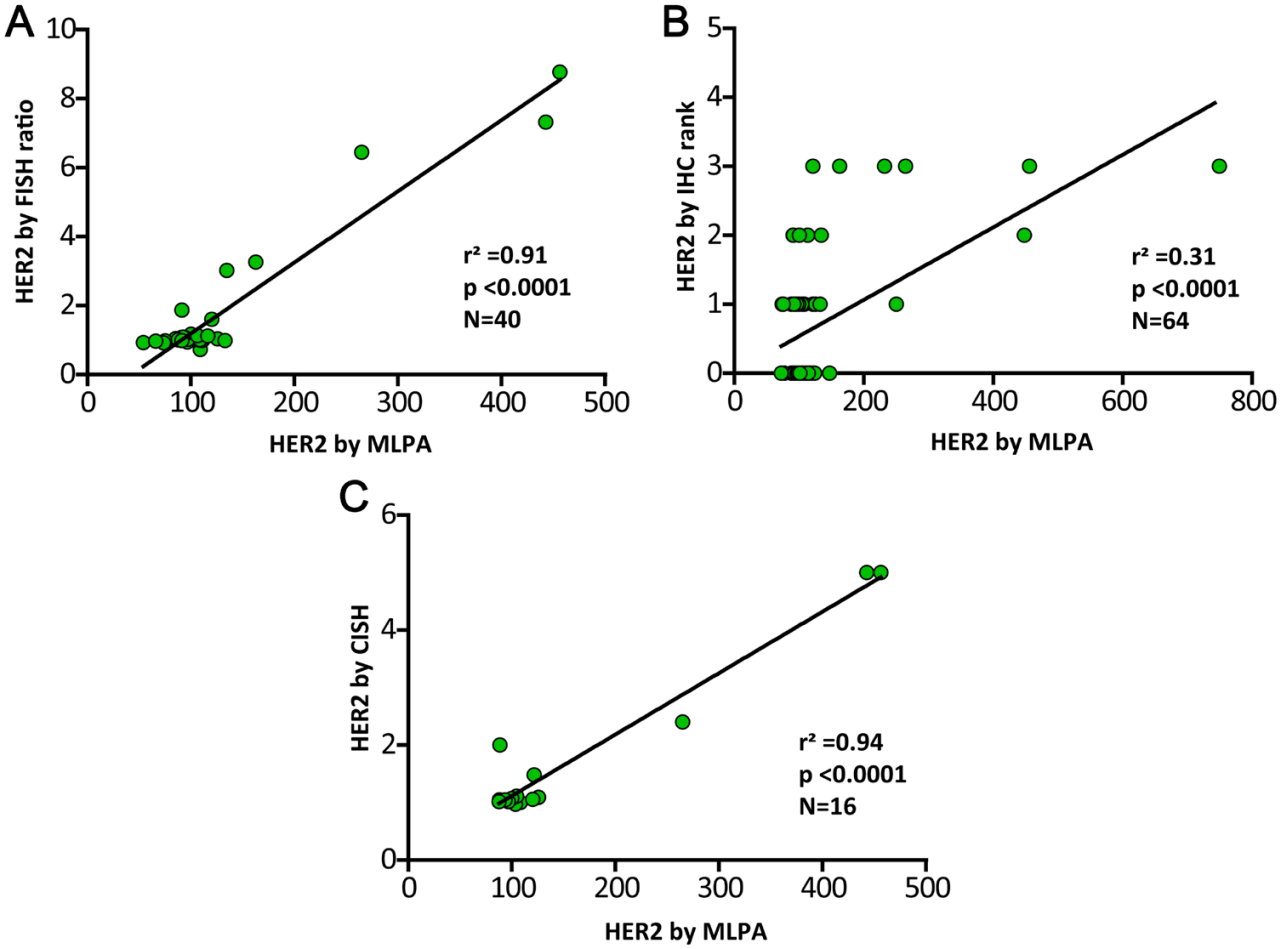
Validation of HER2 MLPA determination by the golden standard procedures FISH, IHC and CISH. Linear regression analyses of the HER2 MLPA results (ratios expressed in percentages) and the three gold-standard determinations. Panel (**A**) for validation by FISH ratios, panel (**B**) by IHQ ranks and panel (**C**) by CISH scalar values. The three approaches reveal significant strong correlation with MLPA.

The validation of the MLPA results for HER2 revealed a positive significant correlation by linear regression analyses with the two gold-standard determinations in both, the prospective and the global analyses, i.e. with FISH ratios (Adjusted *r*^2^ = 0,91, *p* < 0,0001) and with CISH scalar values (Adjusted *r*^2^ = 0,94, *p* < 0,0001) and a medium correlation with IHQ ranks (Adjusted *r*^2^ = 0,31, *p* < 0,0001) (Figure 2). No differences were observed in the correlation analyses between prospective and global study.

Further on, to assess the capacity of the MLPA assay to differentiate between HER2 positive and negative tumor samples, Receiver Operating Characteristic (ROC) curve analyses were performed, using the IHC data as an independent classifier. In this case, the prospective study presented the highest AUC = 1, while when including the retrospective samples, the AUC descended slightly to AUC = 0,966 (95% CI:0.919–1).

To also validate MLPA by real-time quantitative PCR (RT-qPCR), we selected, in addition to tumor samples, some cell lines which were known by the literature to be positive (BT474) and negative (MDA-MB231, Hela) for HER2 expression. Even though the number of included samples was reduced (*N* = 18) due to poor RNA quality and/or available amount of tumor tissue, the results revealed a positive significant correlation (Spearman *r* = 0.79, *p* = 0.0002) between the MLPA and RT-qPCR results.

### Establishment of MLPA cut-off values for the four HER members

After the amplification status of the 4 HER were determined by MLPA in 78 IDC samples and 4 cell lines, we aimed to set a cut-off value to classify the samples as positives or negatives. For this, again we used HER2 as reference for which clear cut-offs are established in IHC or FISH assays. Our aim was to define the MLPA cut-off by correlating it with IHC/FISH thresholds for which the treatment response has been established (even if not necessarily matching the suggested threshold ratio of 1.3 for determining copy number increase in any MLPA reaction).

When establishing the HER2 MLPA-ratio cut-off at 1.59, the correlation seen with FISH ratios, IHC ranks and CISH was strongly positive, i.e. FISH ratios (Adjusted *r*^2^ = 0.82, *p* < 0,0001), IHC ranks (Adjusted *r*^2^ = 0.48, *p* < 0,0001) and CISH scalar values (Adjusted *r*^2^ = 0.76, *p* < 0,0001). Also, the ROC curve analyses showed the highest sensitivity/specificity among the prospective samples (AUC = 1) and the global study samples (AUC = 0.899 95% CI 0.721–1). The chosen 1.59 MLPA cut-off corresponded to the upper limit of the 99% CI of the HER2 MLPA data and represented 89.7% of the samples.

**Figure 3:**
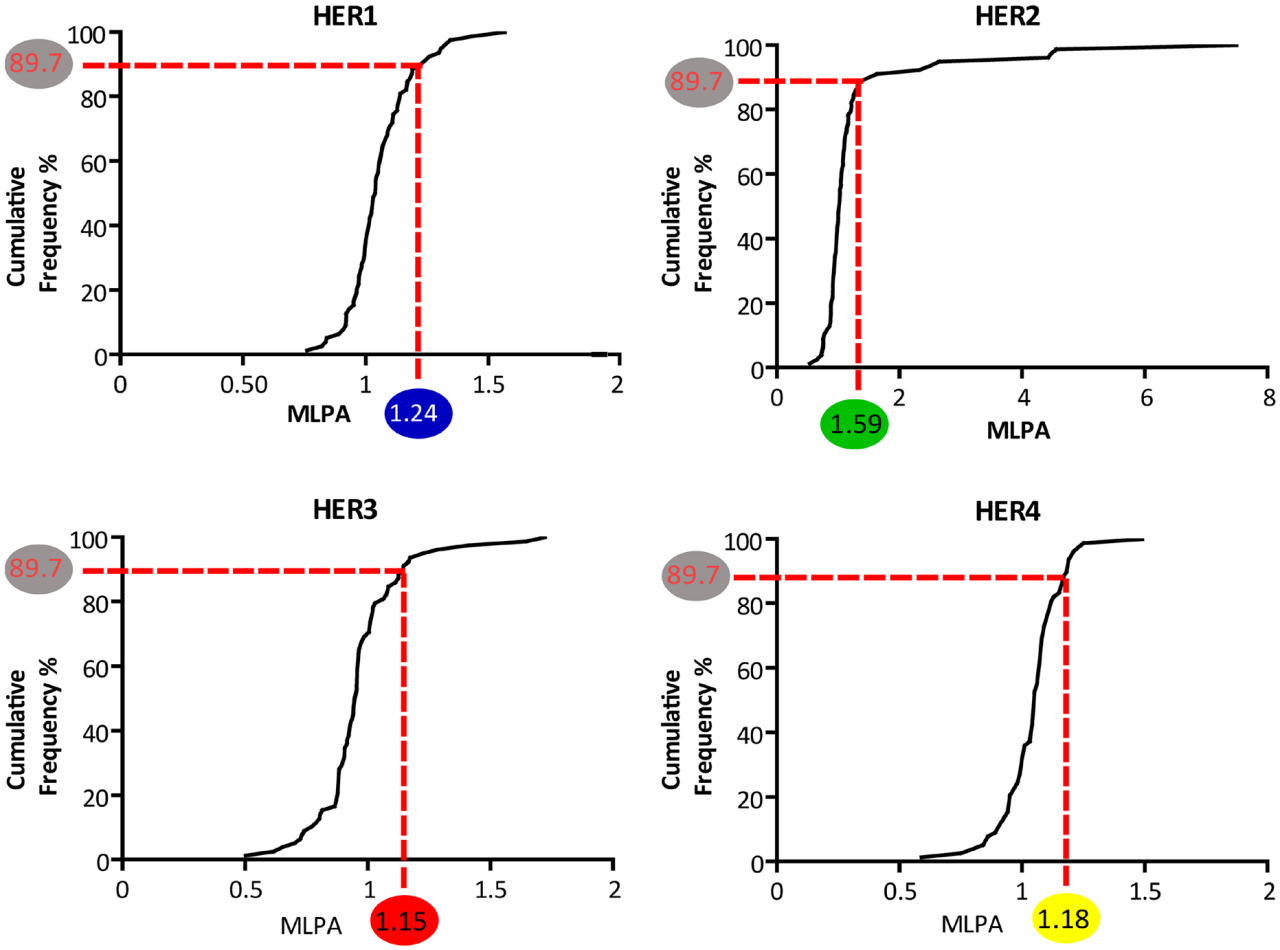
Cumulative frequency distribution of the MLPA probe ratios of HER family gene members. The curves represent the cumulative frequency percent of each HER probes’ mean ratio. In the HER2 panel. the validated MLPA cut-off value (=1.59) is highlighted in a green circle. The corresponding cumulative frequency percentage is 89.7% (highlighted in grey). On the remaining HER1. HER3 and HER4 panels. this percentage has been taken as reference to determine the corresponding MLPA cut-offs. These are: for HER1 = 1.24 (blue circle). for HER3 = 1.15 (red circle). and for HER4 = 1.18 (yellow circle).

To expand the application of MLPA cut-offs to the other HER (which lack of a consented threshold to apply on breast tumors), we choose the values corresponding to the 89.7% of the samples. Given the distribution of the MLPA data varied among the family members (Figure 1) (in line with what others reported in FISH data [16]), we used the cumulative frequency distribution to propose the remaining MLPA cut-off, which were: 1.24 for HER1; 1.15 for HER3; and 1.18 for HER4 (Figure 3).

To verify if the proposed cut-offs were consistent with normal tissue and amplified cell lines (as reported by the literature), three normal margins of surgical resection (M), two normal leucocyte samples (L), and three cancer cell lines (HeLa, T47D and BT474) were analyzed. None of the normal tissues presented values above the proposed cut-offs. In addition, the cell lines presented results in accordance with the literature, i. e. HeLa (HER2 negative), T47D (HER2 negative) and BT474 (HER2 positive). The results of the examined samples are shown in Table 2.

**Table 2:** MLPA results of tumor and control samples

TUMOR	HER1		HER2		HER3		HER4	
	MLPA	Cut-off = 1.24	MLPA	Cut-off = 1.59	MLPA	Cut-off = 1.15	MLPA	Cut-off = 1.18
**FITR1**	0.96	NEG	0.88	NEG	1.01	NEG	1.08	NEG
**FITR2**	0.98	NEG	1.22	NEG	1.08	NEG	1.10	NEG
**FITR3**	1.17	NEG	1.08	NEG	0.92	NEG	1.08	NEG
**FITR4**	**1.26**	**POS**	1.26	NEG	0.91	NEG	1.09	NEG
**FITR5**	0.82	NEG	0.98	NEG	0.88	NEG	1.13	NEG
**FITR6**	1.07	NEG	0.89	NEG	0.96	NEG	1.17	NEG
**FITR7**	0.99	NEG	1.05	NEG	0.99	NEG	1.07	NEG
**FITR8**	1.05	NEG	0.88	NEG	**1.72**	**POS**	**1.49**	**POS**
**FITR9**	1.05	NEG	0.96	NEG	0.87	NEG	1.07	NEG
**FITR10**	1.07	NEG	1.09	NEG	**1.22**	**POS**	**1.19**	**POS**
**FITR11**	1.04	NEG	1.00	NEG	1.06	NEG	1.05	NEG
**FITR12**	0.91	NEG	0.95	NEG	1.14	NEG	1.16	NEG
**FITR13**	0.89	NEG	0.93	NEG	0.92	NEG	1.01	NEG
**FITR15**	1.02	NEG	1.03	NEG	0.95	NEG	0.93	NEG
**FITR16**	1.07	NEG	1.05	NEG	1.02	NEG	**1.19**	**POS**
**FITR17**	1.14	NEG	1.08	NEG	1.13	NEG	**1.21**	**POS**
**FITR18**	0.97	NEG	0.94	NEG	1.02	NEG	1.04	NEG
**FITR19**	1.02	NEG	1.14	NEG	0.90	NEG	0.95	NEG
**FITR20**	0.92	NEG	0.95	NEG	1.09	NEG	1.17	NEG
**FITR21**	1.06	NEG	1.03	NEG	0.96	NEG	1.11	NEG
**FITR22**	0.96	NEG	0.92	NEG	0.88	NEG	1.01	NEG
**FITR23**	1.03	NEG	1.00	NEG	0.90	NEG	1.08	NEG
**FITR24**	1.22	NEG	1.12	NEG	0.77	NEG	1.12	NEG
**FITR25**	0.97	NEG	0.97	NEG	0.95	NEG	1.07	NEG
**FITR26**	0.93	NEG	0.88	NEG	0.88	NEG	1.07	NEG
**FITR27**	0.92	NEG	0.77	NEG	0.92	NEG	1.10	NEG
**FITR28**	0.98	NEG	1.20	NEG	1.02	NEG	**1.25**	**POS**
**FITR29**	1.14	NEG	**2.65**	**POS**	0.96	NEG	1.16	NEG
**FITR30**	1.01	NEG	0.91	NEG	1.01	NEG	1.05	NEG
**FITR31**	0.97	NEG	0.92	NEG	0.94	NEG	1.05	NEG
**FITR32**	1.03	NEG	0.91	NEG	1.01	NEG	1.11	NEG
**FITR33**	1.11	NEG	1.05	NEG	0.97	NEG	1.05	NEG
**FITR34**	1.19	NEG	1.11	NEG	0.94	NEG	1.08	NEG
**FITR35**	0.99	NEG	1.09	NEG	0.96	NEG	**1.21**	**POS**
**FITR36**	**1.44**	**POS**	0.93	NEG	0.96	NEG	1.05	NEG
**FITR37**	1.00	NEG	**4.56**	**POS**	0.94	NEG	1.00	NEG
**FITR38**	**1.24**	**POS**	1.05	NEG	0.80	NEG	1.04	NEG
**FITR39**	**1.33**	**POS**	**4.43**	**POS**	**1.17**	**POS**	**1.23**	**POS**
**FITR40**	1.00	NEG	0.88	NEG	0.88	NEG	1.04	NEG
**FITR41**	1.09	NEG	0.81	NEG	0.82	NEG	1.08	NEG
**FITR42**	1.05	NEG	0.76	NEG	0.89	NEG	1.05	NEG
**FITR43**	1.06	NEG	0.97	NEG	0.91	NEG	1.06	NEG
**FITR44**	0.97	NEG	0.92	NEG	0.96	NEG	1.00	NEG
**FITR45**	0.92	NEG	0.85	NEG	0.96	NEG	1.01	NEG
**FITR46**	1.13	NEG	1.06	NEG	0.93	NEG	1.12	NEG
**FITR47**	**1.31**	**POS**	1.16	NEG	0.73	NEG	**1.18**	**POS**
**FITR48**	1.06	NEG	0.75	NEG	0.79	NEG	0.89	NEG
**FITR49**	1.01	NEG	1.33	NEG	1.17	POS	0.80	NEG
**FITR50**	1.02	NEG	1.63	POS	1.13	NEG	0.93	NEG
**FITR51**	0.76	NEG	0.74	NEG	0.96	NEG	0.75	NEG
**FITR52**	1.04	NEG	1.35	NEG	0.88	NEG	1.05	NEG
**FITR53**	1.02	NEG	0.88	NEG	0.65	NEG	1.05	NEG
**FITR54**	0.95	NEG	0.54	NEG	0.50	NEG	1.19	POS
**FITR55**	1.17	NEG	0.91	NEG	0.73	NEG	1.00	NEG
**FITR56**	0.95	NEG	0.66	NEG	0.94	NEG	0.98	NEG
**FITR57**	1.11	NEG	1.29	NEG	0.62	NEG	0.96	NEG
**FITR58**	1.17	NEG	1.00	NEG	0.88	NEG	0.86	NEG
**CMT38**	1.00	NEG	0.99	NEG	0.91	NEG	1.08	NEG
**CMT45**	0.92	NEG	0.73	NEG	0.70	NEG	1.04	NEG
**CMT46**	1.18	NEG	1.03	NEG	0.96	NEG	0.99	NEG
**CMT47**	1.04	NEG	1.10	NEG	1.03	NEG	0.95	NEG
**CMT54**	1.19	NEG	0.76	NEG	**1.41**	**POS**	0.85	NEG
**CMT33**	1.09	NEG	1.22	NEG	1.11	NEG	1.09	NEG
**CMT53**	0.84	NEG	**2.32**	**POS**	0.87	NEG	1.15	NEG
**CMT135**	1.09	NEG	1.17	NEG	0.97	NEG	0.95	NEG
**CMT20**	1.00	NEG	1.01	NEG	1.01	NEG	1.03	NEG
**CMT31**	1.10	NEG	**4.48**	**POS**	0.74	NEG	0.93	NEG
**CMT93**	0.84	NEG	**2.51**	**POS**	0.81	NEG	1.07	NEG
**CMT179**	1.14	NEG	1.11	NEG	0.98	NEG	0.59	NEG
**CMT178**	1.13	NEG	0.98	NEG	0.88	NEG	0.93	NEG
**CMT150**	**1.57**	**POS**	1.17	NEG	1.08	NEG	0.84	NEG
**CMT151**	1.03	NEG	1.24	NEG	**1.15**	**POS**	1.00	NEG
**CMT152**	1.11	NEG	1.47	NEG	**1.28**	**POS**	0.95	NEG
**CMT165**	1.03	NEG	1.04	NEG	0.96	NEG	0.94	NEG
**CMT167**	1.00	NEG	0.99	NEG	0.93	NEG	0.99	NEG
**CMT168**	1.04	NEG	1.15	NEG	0.96	NEG	0.98	NEG
**CMT170**	**1.30**	**POS**	1.02	NEG	0.93	NEG	1.06	NEG
**CMT124**	**1.35**	**POS**	**7.49**	**POS**	**1.65**	**POS**	1.09	NEG
**CONTROL**	**HER1**		**HER2**		**HER3**		**HER4**	
**Negative**	MLPA	Cut-off = 1.24	MLPA	Cut-off = 1.60	MLPA	Cut-off = 1.15	MLPA	Cut-off = 1.18
**M1**	1.18	NEG	1.51	NEG	1.01	NEG	0.88	NEG
**M2**	0.99	NEG	1.18	NEG	0.95	NEG	1.03	NEG
**M3**	0.90	NEG	0.82	NEG	0.77	NEG	0.75	NEG
**L1**	0.98	NEG	0.98	NEG	1.01	NEG	1.02	NEG
**L2**	1.03	NEG	1.03	NEG	1.00	NEG	0.99	NEG
**Positive**								
**Hela**	**1.38**	**POS**	1.33	NEG	0.95	NEG	0.59	NEG
**T47D**	1.18	NEG	0.98	NEG	**1.31**	**POS**	0.80	NEG
**BT474**	**1.35**	**POS**	**6.67**	**POS**	0.74	NEG	0.87	NEG

MLPA results expressed as ratios (sample/control) and dichotomized in positives/negatives are shown for the 78 IDCs studied (tumor samples labeled as FITR or CMT) in the upper table. In the lower table MLPA results of negative and positive control samples are shown. M = normal margin of surgical resection, L = normal leucocytes. In bold, the results above the established cut-off value are highlighted. None of the negative control samples present values above the cut-offs. All the positive control samples present at least the value of one HER above the cut-off.

With the proposed cut-offs, some tumors revealed ratios above threshold in more than one HER. For example, tumors FITR8 and FITR10 show HER3 and HER4 amplification, tumor CMT124 presents HER1 and HER2 and HER3 amplification and in tumor FITR39 the all four HER appear amplified.

### Validation of MLPA based HER1,3 and 4-determination by RT-qPCR

To validate the MLPA results of the other members of the HER family, we choose RT-qPCR.

**Figure 4:**
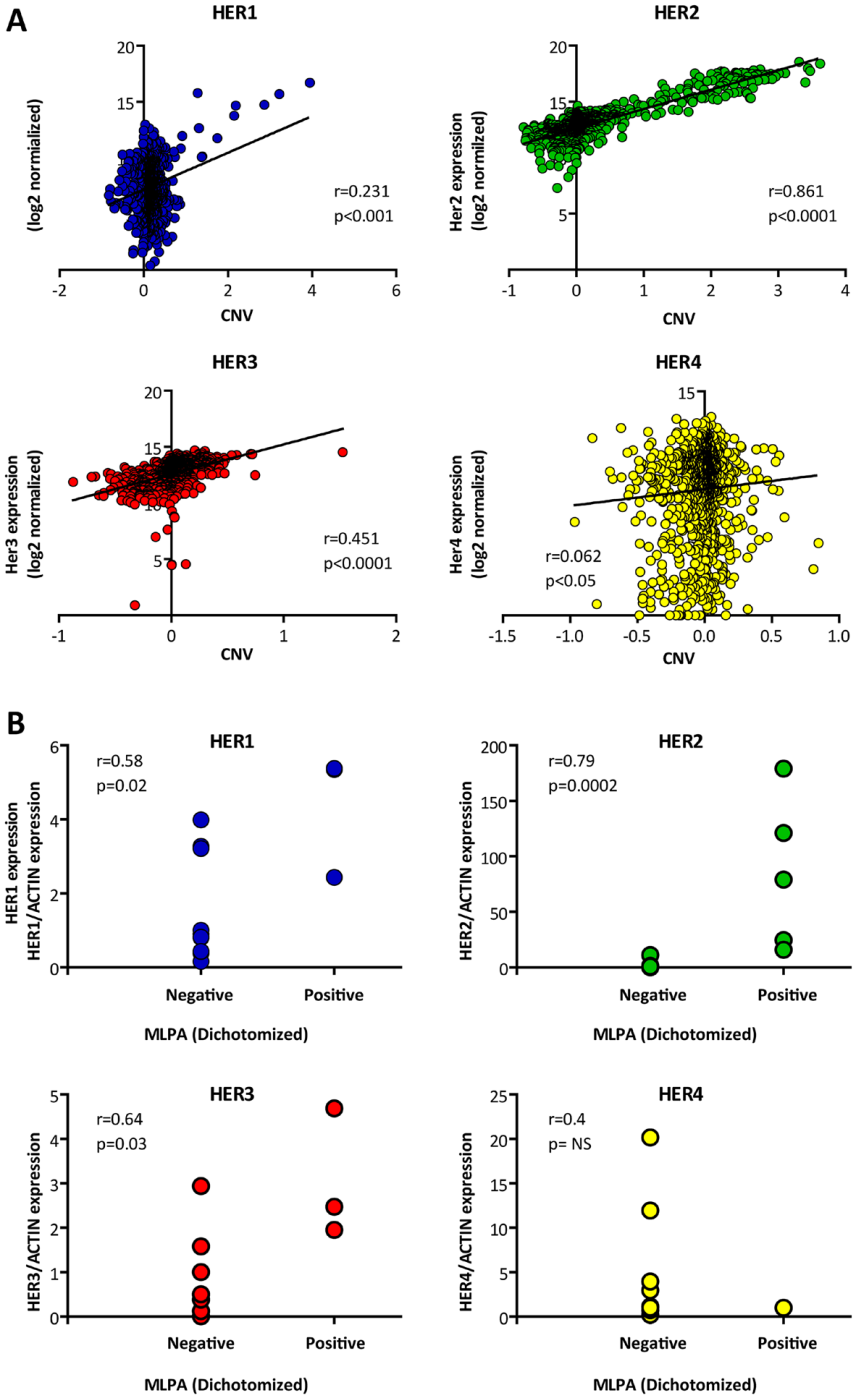
Correlation between gene amplification and expression. (**A**) *In-silico* data from 1095 TCGA IDCs was analyzed to correlate gene amplification (determined by NGS) and expression (determined by RNAseq). As can be observed, the 4 HER present significant positive correlation, determined by Pearson test (*p* < 0.05). (**B**) Wet experiments correlating gene amplification (determined by MLPA) with expression (determined by RT-qPCR) for each HER. The MLPA data was dichotomized based on the previously established cut-offs. The HER expression was normalized to a housekeeping (β-Actin). The number of tested samples were for HER1 = 14. for HER2 = 17. for HER3 = 11. and for HER4 = 10. HER1. HER2 and HER3 present significant positive correlation. determined by Spearman rank test (*p value*s < 0.03).

To this end, we first had to verify the positive correlation between copy number (CN) and gene expression. We therefore performed *in-silico* correlation analyses on 1095 breast tumors from the TCGA Breast Cancer dataset. We found that CN (determined by NGS) and gene expression (determined by RNAseq) presented the strongest correlation coefficients for HER2 and HER3 (i.e. Pearson *r* = 0.861, *p* < 0.0001 and *r* = 0.451, *p* < 0.0001 respectively), followed by HER1 (Pearson *r* = 0.231, *p* < 0.001) and HER4 (Pearson *r* = 0.062, *p* < 0.05) (Figure 4A).

Based on the *in-silico* confirmation that CN correlated with gene expression, we proceeded to the *in-vitro* validation of MLPA by qPCR. The same RNAs which had been used to validate HER2-MLPA determinations were processed for HER1, 3 and 4. The results revealed a positive significant correlation between MLPA values ranked in high-low and gene expression for HER1 (Spearman *r* = 0.58, *p* = 0.02) and HER3 (Spearman *r* = 0.64, *p* = 0.03). HER4 did not present significant association, probably due to the limited number of tested samples and in line with the weak r coefficient seen in the *in-silico* analyses (Figure 4B).

### Determination of HER amplification by MLPA in an independent IDC cohort

After the MLPA validation and cut-off establishment, we further aimed to determine the amplification frequency of the four HER in a blind new fresh-tumor cohort. MLPA analyses were performed on 33 new IDCs and results were dichotomized into *positives* and *negatives*. The most frequently amplified gene found was HER3 (9%, CI 99%: 2.3–29.5), followed by HER2 (6%, CI 99%:1.2–25.6) and HER1 (6%, CI 99%:1.2–25.6). HER4 was found amplified in only one tumor (3%, CI99%:0.3–21.4). Even though the confidence intervals are wide because of the sample size, the observations reveal that even in a reduced number of tumors it was enough to detect the amplification of the four HER members. In one tumor even co-amplification of HER3 plus HER4 was observed.

**Figure 5:**
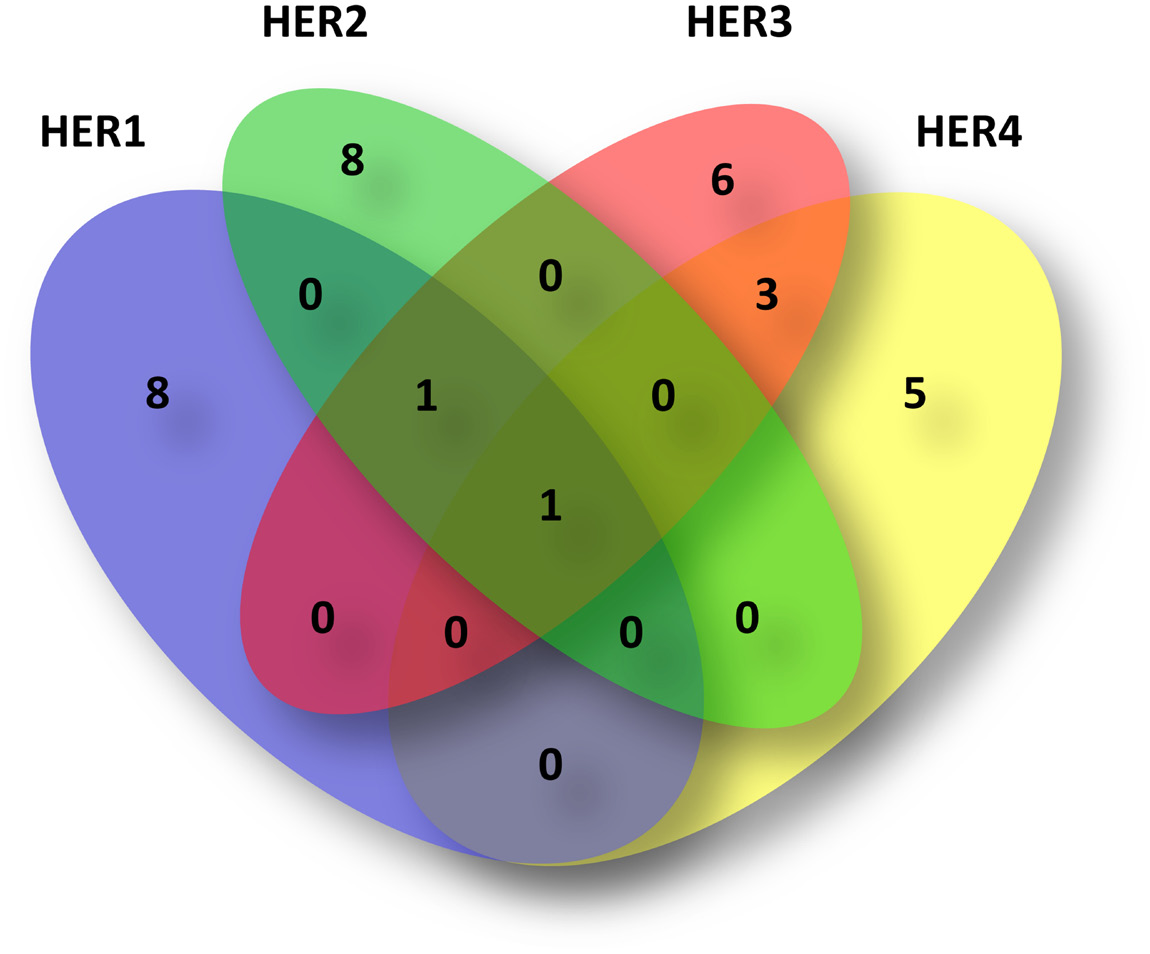
Venn diagram of HER amplified tumors. Of the 111 total IDC included in this work, 32 (28.82%) presented at least one amplified HER as shown in the diagram. Of the 32, five (15.62%) showed co-amplification with another HER member. As can be observed, 27/32 presented individual amplifications, i. e. eight of HER1 (blue circle), eight of HER2 (green circle), six of HER3 (red circle) and five of HER4 (yellow). Among the HER2 amplified tumors, one presented co-amplification with HER3 and one with HER1, HER3 and HER4. Of the HER3 amplified tumors, three presented co-amplification with HER4. HER1 presented in one tumor co-amplification with HER3 and in another tumor with HER2, HER3 and HER4.

Taking together all the included tumors in this work (*N* = 111), 32 (28.82%) presented amplification of at least one of the HER, of which five (15.62%) showed co-amplifications of two or more family members (Figure 5).

### Determination of HER co-amplification by MLPA-ddPCR

The widest application of ddPCR in cancer is for the detection of oncogenic mutations in circulating tumor cells or cell-free tumor DNA [17]. New applications, however, have emerged to assess the level of intra-tumoral heterogeneity [18]. For this work, we aimed to use ddPCR to determine in a tumor sample how many cells presented HER2 co-amplified with another HER member. The standard MLPA results had indicated that in some tumors, more than one HER was amplified. But, as the MLPA assay starts from a DNA sample obtained from many different cells, the result represents only an average of what is going on in the whole tumor. So, we reasoned that a tumor showing by MLPA e.g. HER1 and HER2 amplification, could be due to a 100% of cells with HER1-HER2 co-amplification, 50% of cells with solely HER1 amplification and 50% with solely HER2 amplification, or any combination in-between. To thus determine if the amplifications were occurring in the same cell, we considered to deepening the study at a cellular level.

With this in mind, we reasoned that during the ddPCR assays we needed to maintain the integrity of cells, avoiding lysing membranes and mixing their DNAs. We used the Bio-Rad droplet-digital-PCR platform which is the most widely used in cancer research [19]. After separating cells from FFPE tissues, approximately 2000 were introduced in 20.000 droplets (Supplementary Figure 2) leaving so enough empty droplets needed for the posterior quantification by Poisson equation. Given that the Bio-Rad Platform can detect two different colored targets simultaneously and since HER2 is the favorite partner of the family to heterodimerize with, we designed the Taqman probe for HER2 with HEX (green) fluorescence and gave all the other members the same Taqman fluorescence FAM (blue). So, the multiplexing was performed always assessing the combination of HER2 (green) with one of the other HER (blue).

**Figure 6:**
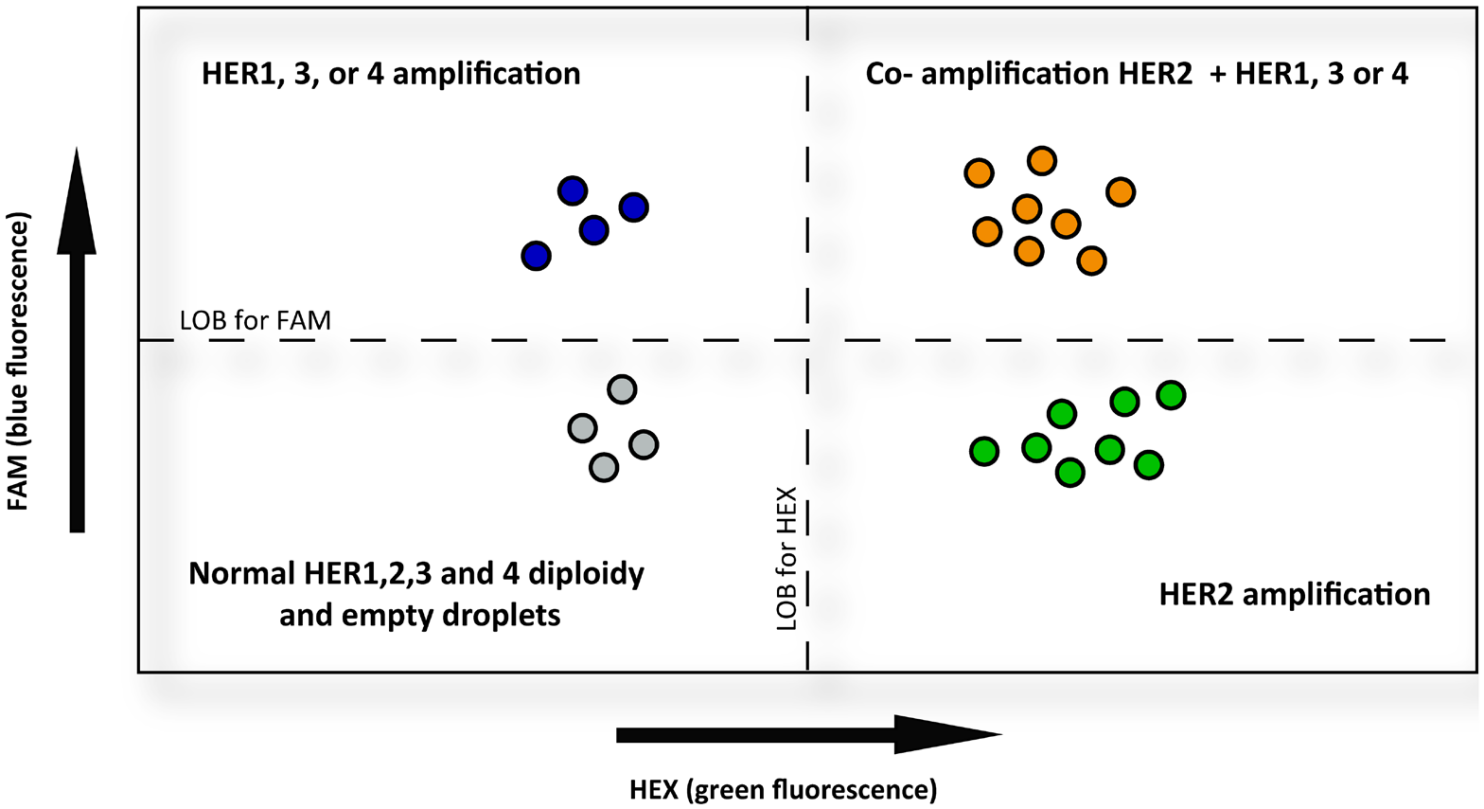
Schematic representation of HER2 co-amplification determined by MLPA-ddPCR. The diagram shows in the horizontal axis the HEX (green) fluorescence and in the vertical axis the FAM (blue) fluorescence. Droplets are represented by colored circles. They contain a tumor cell, were MLPA probes have been previously hybridized to genomic DNA and afterwards labeled Taqman probes are hybridized to a MLPA probe and amplified. HEX-labeled probes were designed to detect HER2 copies, whereas FAM-labeled probes were designed for the remaining HER. In this way, the MLPA-ddPCR assays were performed to determine the co-amplification of HER2 with any of the other HER. The color of each droplet comes from the Taqman probes. Grey colored circles in the left-bottom quadrant indicate normal fluorescence corresponding to diploid cells or basal fluorescence from empty droplets. Green circles in the right-bottom quadrant represent cells with increased HEX fluorescence, indicating HER2 amplification. The more to the right, more increased the fluorescence signal, revealing more HER2 copies. Blue colored circles in the left-upper quadrant represent cells with increased FAM fluorescence, indicating HER1, HER3 or HER4 amplification, depending on the probe used. The higher, the increased fluorescence signal, the more HER1, HER3, or HER4 copies. And finally, the orange droplets at the right-upper quadrant represent cells with both, HEX and FAM increased fluorescence. The number of droplets in this quadrant are indicative of the amount of HER2 co-amplification with the remaining HER. LOB = limit of blank.

We established the LOB limit to include empty droplets plus the fluorescence level of normal diploid cells, which could be part of the tumor population. In this way, droplets with solely HEX or FAM fluorescence above the LOB limit would be indicating cells with one amplified HER. And droplets with merged fluorescence (HEX+ FAM = orange) above the LOB limit, would be indicative of co-amplified HERs in the same cell (see scheme in Figure 6).

**Figure 7:**
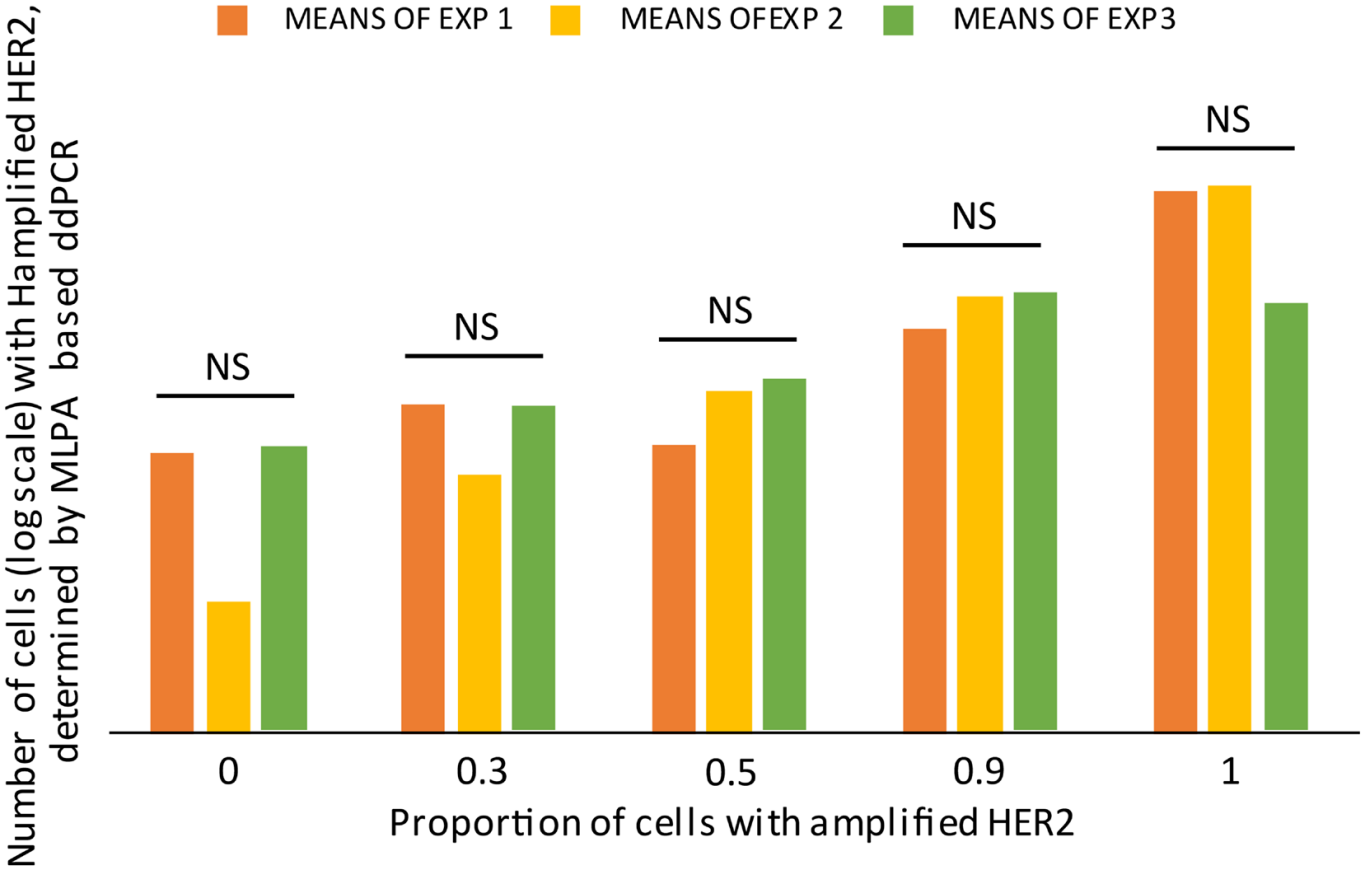
Validation of the MLPA based-ddPCR protocol. Mimic-samples where generated, by mixing HER2-amplified cells (confirmed by standard MLPA) + normal cells in five different proportions. The labeled used refer to the number of HER2-amplified cells and were as follows: 0%, 30%, 50%, 90% and 100%. The means of two technical duplications are plotted in logarithmic scale for three experimental repetitions (EXP1, EXP2, and EXP3). The experimental repetitions for each proportion do not present statistical differences (NS) (Paired *T* test, *p* > 0.04).

To test whether the developed MLPA-based ddPCR protocol was able to reliably quantify the number of cells with an amplified gene, we artificially generated 5 cell-mixtures with the following proportions of HER2 amplified cells: 0%, 30%, 50%, 90% and 100%. Three experimental repetitions were performed, each with technical duplications. To determine the number of cells with amplified HER, we relativized the positive droplets to the total amount of accepted droplets in each experiment. We confirmed that the protocol produced consistent and repeatable results, as can be seen in Figure 7. The three experimental repetitions did not present statistical differences among the same tested proportion (Paired *T* test, *p* > 0.05). And the increasing number of detected HER2-amplified cells is consistent with the augmented proportions.

**Figure 8:**
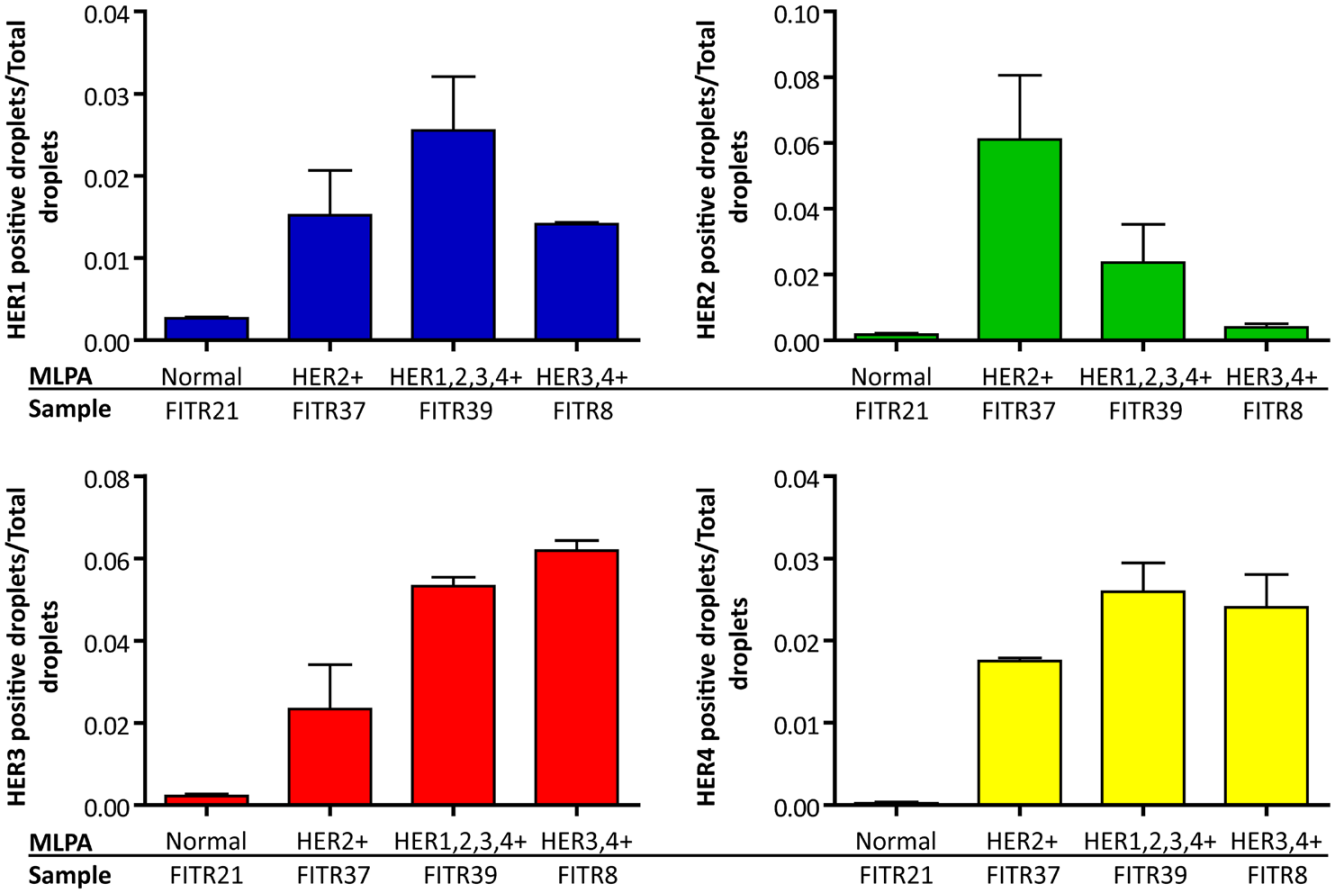
HER copy number determination by MLPA-ddPCR. Gene copy number was determined as a ratio between the number of positive droplets and the total amount of analyzed droplets. Each histogram represents the ratios of a single HER in different tumor samples previously studied by standard MLPA. The analyzed tumors were FITR21 (normal diploid for the four HER). FITR37 (HER2+). FITR39 (HER1, HER2, HER3 and 4+) and FITR8 (HER3, HER4+). We show how HER1 (blue panel) was increased in the only tumor with positive MLPA result (FITR39). HER2 (green panel) was increased in the two tumors with positive MLPA result (FITR37 and FITR39) and FITR37 presented almost a double amount of HER2 positive cells as compared to FITR39 (observation which had not been determinable by standard MLPA). HER3 (red panel) resulted enhanced in FITR8 and FITR39, in line with standard MLPA results. HER4 (yellow panel) showed the highest levels in FITR8 and FITR39 (in accordance with standard MLPA) and an increased amount in FITR37 which was probably not enough to be detected by standard MLPA.

Consequently, we selected tumor samples of our study which had presented by MLPA different HER-status combinations: FITR21 (negative for all the four HERs), FITR37 (HER1, HER3 and HER4 negative, HER2 positive), FITR39 (positive for all the four HERs) and FITR8 (HER1 and HER2 negative, HER3 and HER4 positive) (see details in Table 2). When quantifying each HER by this protocol, we saw the results were consistent with the standard MLPA results, as is shown in Figure 8.

**Figure 9:**
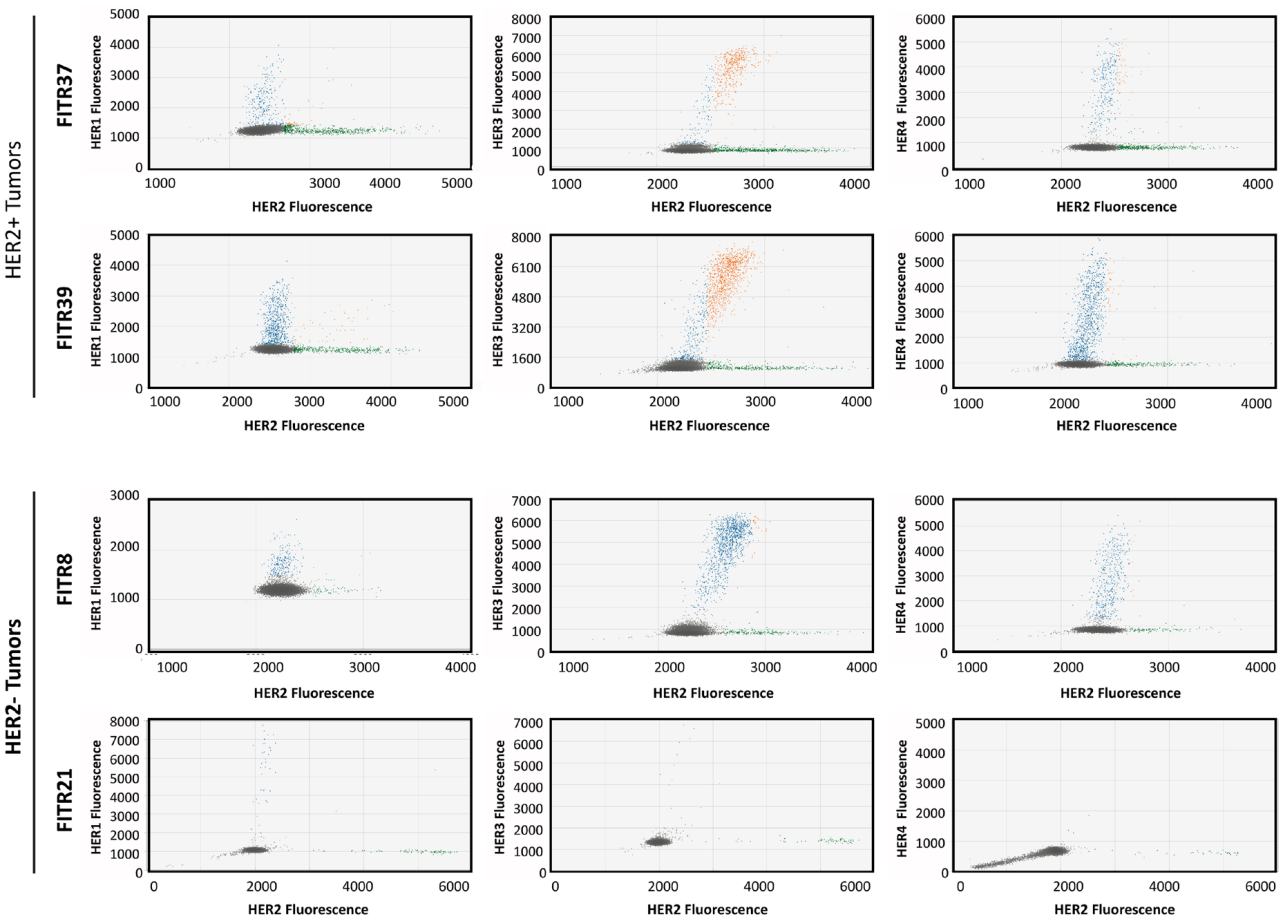
Co-amplification of HER2 with other family members determined by MLPA-ddPCR. Each panel shows the fluorescence amplitude of HEX (HER2) on the X axis vs FAM (other HER) on the Y axis. Dots represent fluorescence of Taqman probes hybridized to MLPA probe, in one cell contained in a droplet. Diploid cells and empty droplets are represented as grey dots. Cells with increased HER1, HER3 or HER4 copies are represented in blue dots and cells with amplified HER2 are represented in green. Cells with co-amplified genes (containing both, HER2 plus HER1, HER3 or HER4 amplifications) are shown as orange dots (see more details in Table 3). As can be seen, in the HER2+ tumor panels, higher co-amplification percentages are observed especially in FITR39. In the HER2- tumor panel, FITR8 (HER3 and HER4 positive, determined by standard MLPA) presents very low co-amplification percentages of HER2 with other HERs since HER2 is not considered amplified by standard MLPA. In FITR21 no amplification is observed.

In Figure 9, images of MLPA-based ddPCR assays are shown for the two HER2 positive tumors (FITR37 and FITR39) and the two HER2 negative tumors (FITR21 and FITR8). The co-amplified percentage of HER2 with another HER are calculated in the 4 tumors as: FAM^+^+HEX^+^ droplets/HEX^+^droplets (Bold droplets/Bold Italic droplets + Bold droplets) (Table 3). We observed that HER3 appears as the more frequent co-amplification partner of HER2. In FITR37, among all the HER2+ cells, 30% are co-amplified with HER3; in FITR39 this percentage ascends to 73%. It is worth to notice that even though FITR37 showed the highest amount of HER2 amplified cells, the co-amplification percentages with other HER is less than expected. On the contrary, FITR39 -with a lower amount of HER2 amplification (as shown in Figure 9) presents the highest co-amplification percentage with HER3 (73%), with HER1 (20%) and with HER4 (13%). Two tumors lacking HER2 amplification were selected as controls (FITR8 and FITR21). Even though some amplifications and co-amplifications are detected (such as HER3 in FITR8, co-amplified with HER2), the percentages are significantly lower as those detected in HER2 positive tumors.

**Table 3:** MLPA-based ddPCR results in four tumor samples

SAMPLE	EXPERIMENT	Italic Positive droplets (mean)	Bold Italic Positive droplets (mean)	Bold Positive droplets (mean)^*^	Bold Positive droplets (%)^**^
**FITR37**	*HER1* **vs*HER2***	239	1020	94	9.21
	*HER3* **vs*HER2***	320	810.5	242	29.85
	*HER4* **vs*HER2***	289.5	431	53.5	12.41
**FITR39**	*HER1* **vs*HER2***	455.25	113.5	22.75	20.04
	*HER3* **vs*HER2***	914	878.25	638.25	72.67
	*HER4* **vs*HER2***	436.75	120.5	16	13.27
**FITR8**	*HER1* **vs*HER2***	225	73	5	6.84
	*HER3* **vs*HER2***	1070	58.5	2.5	4.27
	*HER4* **vs*HER2***	382.5	69	8	11.59
**FITR21**	*HER1* **vs*HER2***	41	104.5	2.5	2.39
	*HER3* **vs*HER2***	34.5	53.5	1	1.86
	*HER4* **vs*HER2***	3	50	1	2

MLPA based ddPCR experiments were performed in four tumors: two HER2 positives (FITR37 and FITR39) and two HER2 negatives (FITR8 and FITR21). For each tumor, assays targeting HER2 combined with one of the remaining HER was performed with technical replications. The mean number of droplets was calculated from the replicated assays. Italic droplets represent cells with HER1, HER3 or HER4 amplifications, Bold italic droplets represent cells with HER2 amplifications, and Bold droplets are cells with both HER amplified. To calculate the percentage of HER2 co-amplified with another HER, the mean number of Bold droplets was relativized to the mean number of Bold italic droplets plus Bold droplets (which is the total amount of cells with HER2 amplification). ^*^Positive droplets for both HER expressed as the mean of 2 technical replications. ^**^Percentage relative to total amount of HER2 positive droplets (Bold italic+Bold).

These observations show that MLPA-based ddPCR is capable to determine co-amplifications at cellular level and the performed experiments suggest that HER2 positive tumors can be heterogeneous in their co-amplification pattern.

To accomplish whether HER co-amplifications could have an impact on the patient’s outcome, we performed *in-silico* analyses utilizing the Breast TCGA dataset with 1095 IDCs. We selected 116 HER2 positive tumors (with high expression, by setting an arbitrary elevated cut-off value > 14.5) and discarded 6 cases who had not received targeted treatment. For the rest, we assumed that at least most of them had been treated, even though only 56cases presented this information loaded in the TCGA dataset. A multiple regression analysis was run to predict the dependent variable “overall survival time” (OS time) from the predictors HER1, HER3 and HER4 expression as a set. The analysis of variance showed an F-ratio = 2.62 at a significant level (*p* = 0.05), revealing the value of HER members to predict OS in a HER2 scenario. When evaluating their role as individual variables, HER3 resulted a significant negative predictor of OS time (*t* = –2.54, *p* = 0.01), whereas HER4 showed a positive trend (*t* = 1.53, *p* = 0.1). HER1 on its own did not show significance in predicting OS time.

In this dataset we could find that in a HER2+ environment, the overexpression of HER3 has a negative impact on the OS time (in line with previous findings by others [20]), while overexpressing HER4 tends to contribute in the opposite direction (also previously seen by others [20, 21]. These analyses suggest the clinical value of the addition of at least HER3 and HER4 data in the context of HER2 determination.

## DISCUSSION

The high homology in the genomic sequence of the four HER oncogenes suggests that they share a common origin, which probably evolved through gene duplications [10]. The fact that the variation in the number of copies in these genes is more frequent than point mutations, confirms that the sequence is still prone to duplication. Their high homology allowed them to evolve towards a robust network signaling system, resistant to common disturbances based on the redundancy of loops, as outlined in the biological advantage of “Bow-Tie Architecture” systems [22].

HER1 and HER2 are clinically validated targets in several cancers, such as squamous cell carcinoma of the head and neck [23], colorectal [24], breast [25], gastric [26], brain [27], and non–small cell lung cancers [28]. And growing evidence suggests that HER3 will prove to be a clinically relevant target as well [8]. Unfortunately, clinical data reveals that a relevant number of patients develop resistance to either anti-HER1 (Cetuximab) and anti-HER2 (Trastuzumab) treatments [29]. Interestingly, Wheeler et al. [30] observed that when inducing resistance to Cetuximab in NSCL cell lines, the cells acquired an increased expression of the other HER members. The authors discovered that when applying a pan-HER blockage, the cells decreased the proliferation rates and xenograft tumors delayed their growth. In line with this, Jacobsen et al. [21] showed how Pan-HER antibodies are able to block the synergic functioning of HER family receptors HER1, HER2 and HER3 across several different cell lines and in xenografts, not only by down regulating their levels but also by inhibiting compensatory upregulations and downstream signaling. At a functional level, when pan-blocking the HER family, cells enter in apoptosis or stop cell cycle. On the contrary, targeting only one receptor can trigger the increase of the others.

Specifically, in breast cancer it has been well established that dysregulated expression and activity of HER family members is frequent. Overexpression of HER1, HER2 and HER3 is generally associated with poor prognosis whereas high expression of HER4 is associated with a better outcome [20, 21]. In patients, the antitumor activity of dual HER blockade (with trastuzumab in combination with the dimerization inhibitor pertuzumab or the TK inhibitor lapatinib) was proven to be significantly superior to single agents in a neoadjuvant setting [31].

Findings from the randomized phase 3 Neo ALTTO trial in women with HER2-positive early breast cancer showed that the combination of lapatinib and trastuzumab significantly improved rates of pathological complete response compared with either drug alone. Although event-free survival or overall survival did not differ between treatment groups, findings from that study confirm that patients who achieve pathological complete response after neoadjuvant anti-HER2 therapy have longer event-free and overall survival than do patients without pathological complete response [32].

The relevant observations of Sergina et al. [33] on HER2 positive breast tumors are in accordance with the concept of HER family synergic working. They observed that TK inhibition can be by-passed by a compensatory shift to HER3 functioning (which lacks TK activity), and that downregulating HER3 expression restores the response to TK inhibition. However, worth to mention is the Hellenic Cooperative Oncology Group (HeCOG) discordant study, who determined in a retrospective analysis the prognostic value of all four HER family receptors in patients with metastatic breast cancer and found that HER3 overexpression associates with lower risk for death in HER2+ patients [34].

Our *in-silico* findings show how the outcome of HER2+ patients is conditioned by the status of at least HER3 and HER4. Previous work published by Kurozumi et al. [35] has shown the existence of a positive correlation of the mRNA expression of HER2 with other members of the family such as HER1 and HER3. A worth observation regarding these findings, however, is that the expression data are not revealing what is happening at a single cell level. Even though it is reasonable to assume that many cells probably present co-altered HER, we think that more accurate associations with patient’s outcome can emerge if the experiments show data at a single cell level and take in account the cellular heterogeneity composition of the tumors.

It is known that intra-tumor heterogeneity can unfavorably influence responses to anti-HER2 therapy [36]. Sub-populations raised from different clones can present a variety of HER amplification combinations. Malinowsky K et al. [37] have recently shown by reverse-phase-protein arrays that the HER members are heterogeneously expressed in primary breast cancers, with an intra-tumor variation coefficient of approximately 20%. The authors found considerable differences in the amplification of HER family receptors on different tumor zones. It is licit to assume that the proportion of the tumor with different combinations of amplified receptors can be associated with the variable response to treatments and the outcome of the patients.

Taken together, the literature and our *in-silico* findings support the relevance of determining the status all the HER members and the tumor-proportion in which they present co-amplification. Our study proposes the use of MLPA for HER2 amplification determination, as it additionally allows simultaneously detection of copy number status of the other HER members and reliably works on FFPE tissue derived DNA. MLPA is affordable in terms of costs for any molecular laboratory or hospital of less developed countries, it does not require highly trained human resources to interpret expanded genomic NGS data, and the developed probemix focuses exclusively on the HER oncogenes analysis. With this first approach, the status of the four HER can be easily determined in 24 hours. In those cases where, in addition to HER2, another HER member is amplified, the determination of the intra-tumoral heterogeneity can be relevant to later associate with a possible treatment resistance. Our developed algorithm based on a deeper partitioned analysis by MLPA-based ddPCR can reveal the proportion in which the co-amplifications with HER2 are occurring. ddPCR has shown to have high sensitivity and accuracy for compartmentalized tumor screening [38]. The assay is relatively simple, is realizable from FFPE samples and requires the specific equipment for droplets generation and fluorescence reading. The costs are affordable like a FISH determination.

We have validated the MLPA probes by gold standard procedures for HER2 and by RT-qPCR for the rest of the HER family. We have also demonstrated the feasibility of quantifying the co-amplified tumor proportion by MLPA-ddPCR. Our development encourages and facilitates further studies to investigate prospectively the association of co-amplified HERs with patient’s outcome parameters such as overall survival and relapse free survival. It also permits to investigate any relationship between the co-amplified tumor proportion and the patient response to single or combined treatment. Such studies would help to determine whether the assessment of the HER family is a reliable predictive marker for anti-HER2 treatment response and/or combined anti-HER therapies selection. Finally, the observations rising from breast cancer studies could serve as proof of principle for other tumors which commonly present the alteration of HER members.

## MATERIALS AND METHODS

### Tumor samples

Ethical approval was obtained from the Ethics Committee of the Faculty of Medical Sciences of the National University of Cuyo, to perform the study on tumor samples of breast cancer patients who signed an informed consent. A total of 111 IDC samples were included of which: 57 were formalin-fixed and paraffin-embedded (FFPE) and 54 fresh frozen. Samples were non-macrodissected. Tumor content was confirmed by experienced pathologist after surgery and prior to anatomopathological examination. Samples with less than 32% of tumor cells were discarded. All patients had not undergone neoadjuvant treatment and were operated at the Instituto GinecoMamario of Mendoza by the same surgeon. Clinical data of 54 patients was available.

Consecutive serial sections from FFPE tissue were obtained using a microtome. Two 3–5 μm-thin sections were mounted per glass slide. The first two sections were mounted on a regular glass slide for hematoxylin and eosin staining. The next sections were mounted on positively charged slides, one to perform IHC, a second for FISH and a third for CISH, resulting in four slides for each tumor. Additionally, five 10 μm-thin sections were collected in 1.5 ml tube and set aside for DNA and RNA extraction. Finally, two 30 μm-thin sections were collected in 1.5 ml tubes for cell extraction, to perform MLPA based dPCR assays.

### Cell lines

Breast cancer cell lines T47D, MDA-MB231 and BT474 were kindly provided by Dr. Roxana Schillaci from IBYME-CONICET (Argentina) and maintained with a controlled number of passes. The cervical cancer cell line HeLa was provided by Dr. Marisa Colombo from IHEM-CONICET (Argentina). All cell lines were cultured using DMEM (Invitrogen, USA) supplemented with 10% fetal bovine serum (Gibco, USA) and 1% of ampicillin (Invitrogen, USA). Cells were harvested to extract DNA or RNA to use as controls in further studies.

### Immunohistochemistry

Samples were fixed in neutral formalin between 6–48 hours and then embedded in paraffin. Haematoxyline eosin staining was performed using standard histological techniques. For immunohistochemistry (IHC), 4 μm sections were incubated with anti-HER2 4B5 antibody (Ventana Medical Systems, Tucson AZ). Staining procedures were performed on automated Benchmark XT stainer (Ventana Medical Systems, Tucson, AZ) using the Ultraview DAB detection system. Slides were evaluated by a trained pathologist according to ASCO/CAP 2018 guidelines [1]. A case was considered positive (score 3+) if complete and intense circumferential staining was observed in > 10% of tumor cells, equivocal (score 2+) if weak to moderate staining was observed in > 10% de of tumor cells, and negative if faint staining was observed in > 10% (score 1+) or if no staining or faint staining was observed in < 10% of tumor cells. Only invasive carcinoma cells were selected for analysis.

### FISH

FISH procedure was performed on 4 μm sections of FFPE samples using a dual Path Vysion Her2 probe (Abbott Molecular Inc., Downers Grove, Illinois, USA) only invasive carcinoma cells were selected for analysis. At least 50 cells were scored by a trained observer for nuclear HER2 and chromosome CEP17signals. HER2/CEP17 ratio and mean HER2 copy number was obtained for each case. Cases were grouped in the following categories according to ASCO/CAP guidelines: Group 1, HER2/CEP 17 ratio ≥ 2 mean HER2 copies ≥ 4; Group 2 HER2/CEP17 ratio ≥ 2 mean HER2 copies < 4; Group 3 HER2/CEP17 ratio < 2 mean HER2 copies ≥ 6; Group 4 HER2/CEP17 ratio < 2 mean HER2 copies ≥ 4 and < 6; Group 5 HER2/CEP17 ratio < 2 mean HER2 copies < 4. A case was considered HER2 positive by FISH if HER2/CEP17 ratio ≥ 2 mean HER2 copies ≥ 4 and/or if 3 HER2/CEP17 ratio < 2 mean HER2 copies ≥ 6.

### DUAL CISH

Chromogenic hybridization techniques were performed on 4 μm sections using INFORM HER2 Dual ISH DNA Probe Assay using the BenchMark XT Staining platform. All samples were processed following the FDA-approved protocol. Only invasive carcinoma cells were selected for analysis. At least 50 cells were scored for nuclear HER2 and chromosome. CEP17signals. HER2/CEP17 ratio and mean HER2 copy number was obtained for each case. Cases were grouped in categories 1–5 according to ASCO/CAP2018 guidelines as in FISH cases and criteria for HER2 positivity were similar.

### DNA extraction

Genomic DNA was isolated from FFPE tissues following the one-tube-FFPE-extraction protocol reported by Atanesyan L et al. [39]. Briefly, tissue sections obtained as described above were immersed in the SALSA FFPE buffer (50 mM Tris-HCl pH 8.5, 100 mM NaCl, 1 mM EDTA, 0.5% TWEEN 20 and 0.5% NP40), incubated for 15 minutes at 90°C and finally treated with proteinase K at 55°C overnight. DNA from fresh tissues was isolated using CTAB (Cetyltrimethyl Ammonium Bromide) as previously described [40] and DNA from cell lines was extracted using the Pure Link Genomic DNA Mini Kit (Invitrogen) according to the manufacturer’s instructions.

### MLPA

Multiplex Ligation-Dependent Probe Amplification (MLPA) analysis was performed using SALSA MLPA probemix P483-A1 (MRC Holland, Amsterdam, The Netherlands) according to simple protocol originally described in Schouten JP et al. [15]. The probe mix contains six probes for HER1, eight for HER2, four for HER3 and five for HER4, plus eight probes flanking the HER regions and 16 reference probes located on chromosomes were no HERs are located. Reactions were carried out following manufacturer’s instructions. Briefly, all the MLPA probes were hybridized to their complementary sequences in the genomic DNA overnight, followed by ligation of hybridized left and right parts of probes, and further amplification of the ligated probes using fluorescent PCR-primer pair. Finally, fluorescent PCR products were separated by capillary electrophoresis in a Beckman CEQ8000 sequencer (Beckman Coulter Inc. Fullerton, CA, USA) or in an ABI 3130 capillary sequencer (Applied Biosystems, Foster City, CA, USA) and analyzed by the GeneMarker v1.75 software (Softgenetics LLC, PA, USA). As control samples, leukocytes and healthy breast tissue obtained from margins of surgical resection were used. As reference samples should be purified using the same isolation method as the test samples, we used DNA from either formalin-fixed or fresh lymphocytes or surgical margins according to the tissue source to be tested. MLPA results were presented as ratios of normalized fluorescent PCR fragment heights between tumor/control samples.

### RNA extraction and quantitative PCR

RNA was extracted from fresh tumors and cell lines with Trizol Reagent (Life Technologies, USA) and PureLink RNA Mini Kit (Invitrogen, USA). 1 μg of total RNA was used for first strand synthesis of cDNA by using M-MLV retro-transcriptase kit (K1600) (Inbio Highway, Argentina) and Random Hexamers (Roche, USA) primers. A post-isolation DNase treatment was applied after each extraction to avoid DNA amplification in later experiments. The RNA was re-suspended in nuclease-free water and concentrations were estimated by optical density measurement using the Nano Spectrophotometer LNS-101 (Labocon). The reverse transcription reaction was carried out during 60 minutes at 37°C according to manufacturer´s instructions.

Expression levels of the HER genes as well as of the reference gene β-ACTIN and a negative control (lacking cDNA) were assessed in duplicated or triplicated RT-qPCR experiments using the QuantiNova SYBR Green PCR Kit on an AriaMx Real-time PCR System (Agilent Technologies, Germany). Initially, 3 housekeeping genes were tested, i.e. β-ACTIN, B2M and GAPDH, but given the wide dispersion between samples, β-ACTIN was chosen as the more stable to use for expression normalization.

The program used was: 3 min at 94°C followed by 40 cycles of 20 s at 94°C, 15 s at 60°C, and 15 s at 72°C. The sequence of the primer sets used have been previously described by Koutras et al. [34] and are shown in Table 4.

**Table 4:** RT-qPCR primer sequences

	Exon	Forward	Reverse
**HER1**	7	CGCAAGTGTAAGAAGTGCGAA	CGTAGCATTTATGGAGAGTGAGTCT
**HER2**	32	TCTGGACGTGCCAGTGTGAA	CCTGCTCCCTGAGGACACAT
**HER3**	27	CGGTTATGTCATGCCAGATACAC	GAACTGAGACCCACTGAAGAAAGG
**HER4**	6	GAGGCTGCTCAGGACCTAAGG	GAGTAACACATGCTCCACTGTCATT
**β-Actin**	5,6	TGACGTGGSCATCCGCAAAG	CTGGAAGGTGGACAGCGAGG

Sequences of forward and reverse primers used in RT-qPCR experiments, for the four HER oncogenes and the housekeeping gene β-ACTIN.

### MLPA-based droplet digital PCR

Cells from breast cancer FFPE tissues were obtained with a modified protocol described by Corver and Haar [41]. Briefly, 2 × 30 μm tissue sections were deparaffinized with xylol and rehydrated by sequential immersion in decreasing concentrations of ethanol, followed by the incubation with proteinase K buffer (10% proteinase K in RPMI medium) for 40 minutes at 50°C. Afterwards, the suspension was filtered through an 80 μm nylon filter and centrifuged 10 minutes at 2000 rpm, the supernatant was discarded and the cell pellet was resuspended in 50 μl of PBS. The obtained cells were counted with a Neubauer chamber.

In general, the ddPCR assay starts by partitioning the sample into cells and introducing the cells plus a PCR mixture into 20.000 highly uniform droplets through a water-in-oil emulsion. Afterwards, a fluorescent-labeled PCR reaction is performed on the genes of interest inside each droplet, and an absolute quantification of amplified gene copies can be done. Depending on the fluorescence amplitude, droplets are classified as positive or negative using a binary threshold. We considered as positive the droplets that had higher fluorescence value than the fluorescence emitted by the empty droplets or droplets with normal diploid cells. The limit of blank (LOB) was defined as the fluorescence level above which the cells are considered to present amplified signal.

To develop a MLPA-based ddPCR approach, we started with approximately 2000 cells per sample (derived from either cultured cancer cell lines or breast cancer FFPE tissue), to reduce the probability that more than 1 cell entered per droplet. The MLPA probe hybridization and ligation steps were performed with the protocol described above (MLPA section) with subtle modifications (i.e., initial denaturation step time was decreased from 5 minutes to 2 minutes) to minimize cell lysis. Afterwards, 40 μl of probe-ligated cells were centrifuged (3 minutes at 1800 RPM) and resuspended in 10 μl of PBS buffer to eliminate the lysed cells and free DNA. Next, 6 μl with the approximately 2000 cells with ligated probes were enclosed in oil droplets (Droplet Generation Oil for Probes, Bio-Rad Laboratories, USA) together with the PCR mix (ddPCRSupermix for Probes, Bio-Rad Laboratories, USA), un-labeled primers (2 μM) specific for probe amplification and Taq-Man probes (500 μM) designed in our lab to hybridize to one MLPA probe per HER oncogene. HER2 Taqman probe was marked with HEX fluorophore and Taqman probes for the remaining HER oncogenes were marked with FAM fluorophore. To assure that only ligated MLPA probes were detected, the binding site for the designed Taq-Man probes included the ligation site of the MLPA probes (Supplementary Figure 3). The formation of droplets (around 10–18.000) was carried out in 20 μl partition volume in a QX200 Droplet Generator (Bio-Rad Laboratories, USA). The amplification was carried out in a T100 Thermal Cycler (Bio-Rad Laboratories, USA) using the following program: initial denaturation at 95°C for 10 minutes, amplification 60 cycles of 30 seconds at 95°, 70 seconds at 60° and after 60 seconds at 90°. For all steps a ramp rate of 2°C/s was used. Finally, the fluorescence generated in droplets was measured in a QX200 Droplet Reader (Bio-Rad Laboratories, USA). Technical replications were performed for droplet number calculations. The mean number of accepted droplets was 16530, SD = 2691,22 (95% CI: 15836,52–17226,95). Data analyses were performed Using Quantasoft Software Analysis 1.0 (Bio-Rad Laboratories, USA).

### Statistical analyses

Normal distribution of all data was tested by Kolmogorov-Smirnov test. The association between HER2 MLPA results and FISH scores, IHC ranks and CISH scalar data was determined using parametric and non-parametric correlation tests (Pearson and Spearman-rank tests) and linear regression analyses. To validate MLPA HER2 results, the sensitivity and specificity was determined by ROC curve analyses referred to IHC data. The correlation between gene amplification and expression *in-silico* data, and afterwards the validation of MLPA HER1, HER3 and HER4 results by RT-qPCR was determined by Pearson and Spearman-rank test. Prediction of overall survival time from HER status of *in-silico* data was analyzed by multiple regression analyses entering all variables in the model in one single step (Enter Method). All statistical analyses were carried out using GraphPad Prism v5, IBM SPSS Statistics v19 and MedCalc (https://www.medcalc.org/).

### Ethics statement

The project and corresponding informed consent were evaluated and approved by the Ethics Committee of the Faculty of Medical Sciences. National University of Cuyo. Mendoza. Argentina on the 15th of December 2014. References number of the committee: comite_bioetica@fcm.uncu.edu.ar. File number of the approval: 14594/2014.

## SUPPLEMENTARY MATERIALS


