# Supplementary material for: Working together for the family: determination of HER oncogene co-amplifications in breast cancer
Source: Oncotarget. 2020 Jul 14;11(28):2774–92. doi: 10.18632/oncotarget.27671 (PMC7367656; doi:10.18632/oncotarget.27671)
Supplement: Supplementary file 1 [file oncotarget-11-2774-s001.pdf]

Working together for the family: determination of HER oncogene co-amplifications in breast cancer

SUPPLEMENTARY MATERIALS

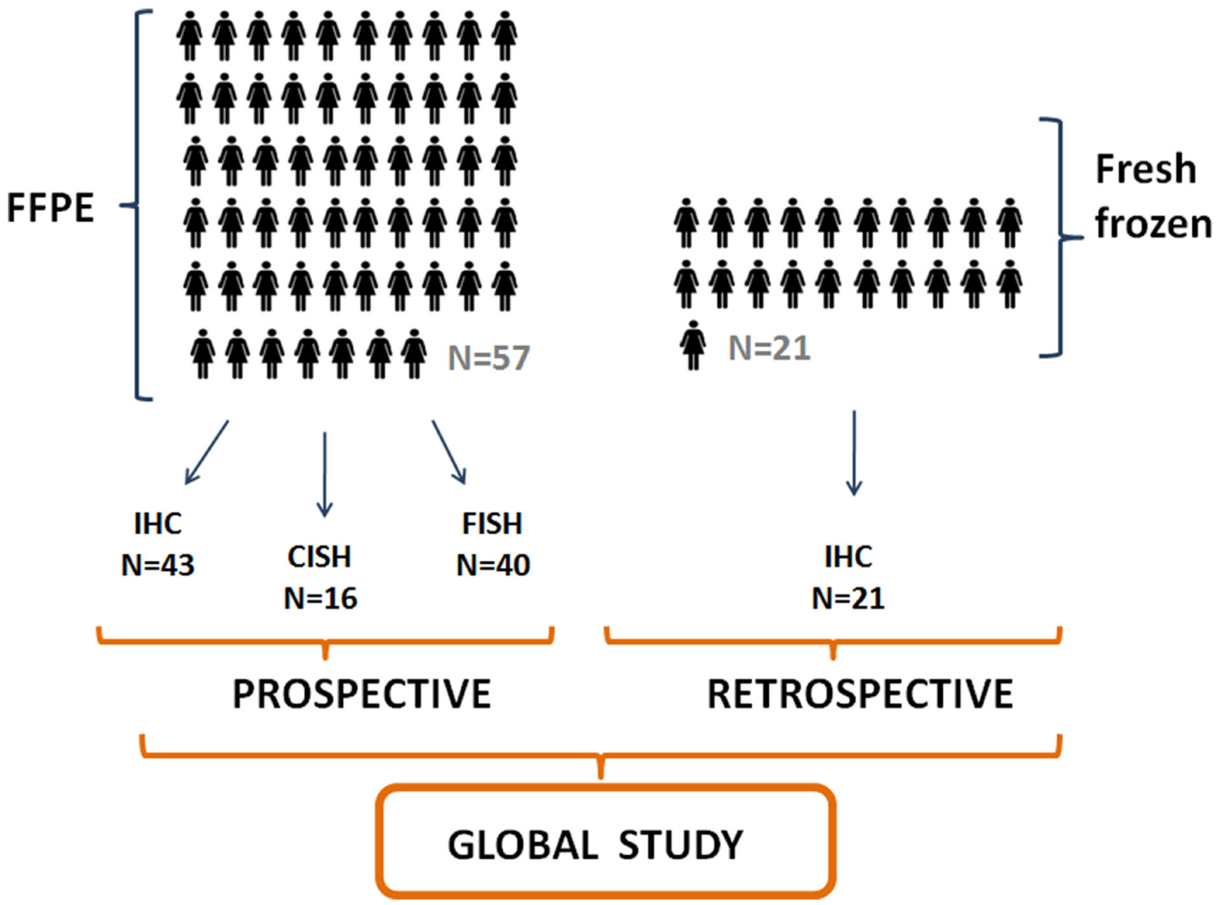

Supplementary Figure 1: Study design and overall strategy for Her family analysis in prospective and retrospective cases.

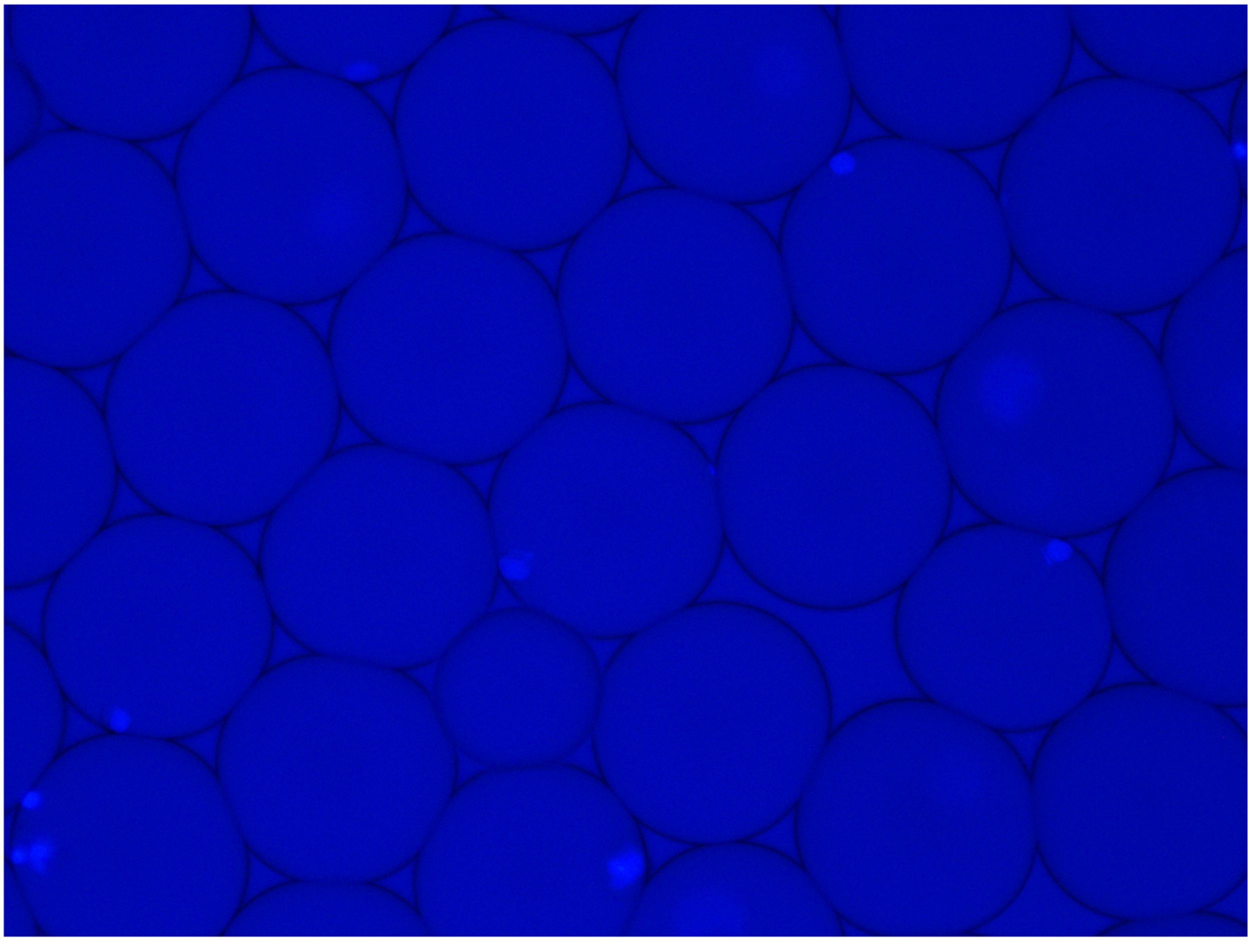

Supplementary Figure 2: Fluorescence microscopy of disaggregated cells included in oil droplets. The cells were stained with DAPI and observed at 20× objective.

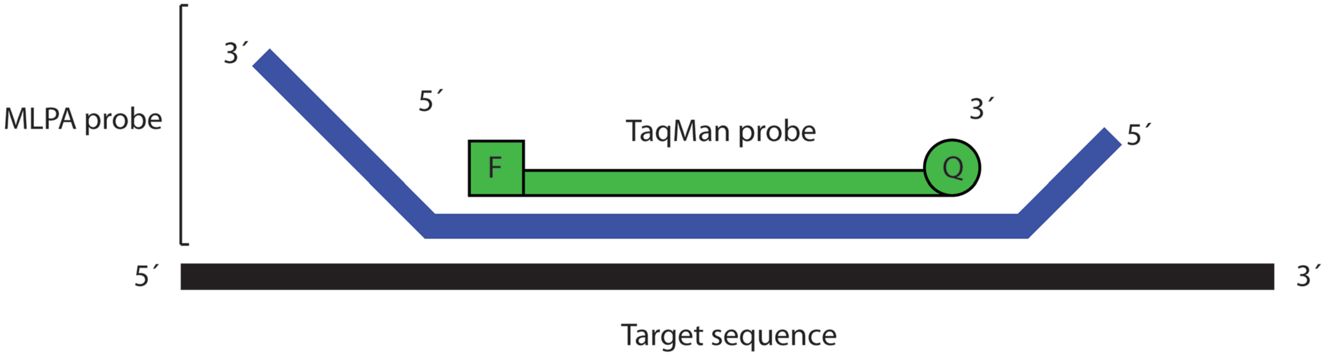

Supplementary Figure 3: Schematic representation of Taqman probes design and site of hybridization with MLPA probes.
